# The importance of early life in childhood obesity and related diseases: a report from the 2014 Gravida Strategic Summit

**DOI:** 10.1017/S2040174414000488

**Published:** 2014-10-13

**Authors:** E. C. Macaulay, E. L. Donovan, M. P. Leask, F. H. Bloomfield, M. H. Vickers, P. K. Dearden, P. N. Baker

**Affiliations:** 1 Gravida: National Centre for Growth and Development, University of Auckland, Auckland, New Zealand; 2 Department of Pathology, Dunedin School of Medicine, University of Otago, Dunedin, New Zealand; 3 The Liggins Institute, University of Auckland, Auckland, New Zealand; 4 Genetics Otago, Department of Biochemistry, University of Otago, Dunedin, New Zealand; 5 Department of Paediatrics: Child and Youth Health, University of Auckland, Auckland, New Zealand

**Keywords:** biomarker, development, intervention, mechanism, obesity, pregnancy

## Abstract

Obesity and its related non-communicable diseases (NCDs), such as type 2 diabetes, heart disease and cancer, impose huge burdens on society, particularly the healthcare system. Until recently, public health and policy were primarily focused on secondary prevention and treatment of NCDs. However, epidemiological and experimental evidence indicates that early-life exposures influence the risk of childhood obesity and related diseases later in life, and has now focused attention on the health of both mother and child. During pregnancy and the early neonatal period, individuals respond to their environment by establishing anatomical, physiological and biochemical trajectories that shape their future health. This period of developmental plasticity provides an early window of opportunity to mitigate the environmental insults that may increase an individual’s sensitivity to, or risk of, developing obesity or related diseases later in life. Although much investigation has already occurred in the area of *Developmental Origins of Health and Disease* research, the science itself is still in its infancy. It remains for researchers to tackle the important outstanding questions and translate their knowledge into workable solutions for the public good. The challenge, however, is to decide which areas to focus on. With these opportunities and challenges in mind, the 2014 Gravida Summit convened to examine how its early-life research program can determine which areas of research into mechanisms, biomarkers and interventions could contribute to the international research strategy to fight childhood obesity and its related diseases.

## Introduction

Global rates of overweight and obesity and their related health conditions have increased rapidly over the last 30 years^1^ and impose large burdens on public health systems, the economy and society.^2^ Worldwide, the prevalence of overweight and obesity combined rose by 27.5% for adults and 47.1% for children between 1980 and 2013.^1^ New Zealand is among those countries with the greatest increase in obesity in the last 30 years, particularly among men;^1^ ∼29% of boys and girls aged <20 years are overweight or obese, as well as ∼71% of adult males and 60% of adult females.^1^ In addition, 65% of women who give birth each year in socioeconomically disadvantaged areas of New Zealand are overweight or obese.^3^

There is increasing evidence that influences during early life are inextricably linked to the later development of obesity and related non-communicable diseases (NCDs), such as type 2 diabetes, hypertension, heart disease and many cancers.^4^ ^–^ ^8^ Developing organisms (including humans) appear to respond to their early-life environment and set anatomical, physiological and biochemical trajectories that shape their future life course.^9^ ^–^ ^13^ This period of developmental plasticity appears to involve not only the period from conception to birth, but also the very early years after birth.^9^ ^,^ ^14^ ^,^ ^15^ These windows early in an individual’s life provides a unique opportunity to reduce or mitigate the cues that later increase their sensitivity to, or risk of, developing obesity or NCDs later in life.^16^

Maternal condition and environmental factors during gestation can influence obesity and related metabolic diseases in early adulthood.^17^ ^,^ ^18^ For example, low maternal vitamin B_12_ and high red cell folate in pregnancy predicted increased insulin resistance in offspring at 6 years of age.^19^ Maternal adiposity, greater gestational weight gain and parity all impact the adiposity of the offspring.^20^ Maternal obesity also has marked effects on the structure and function of the placenta,^21^ demonstrating that maternal nutrition plays a critical role in programming foetal development. However, early-life exposures generally do not directly influence pathophysiology. Rather, they alter the sensitivity of the organism and the organism’s response to subsequent environmental cues, which can be pathogenic.^22^

Until recently, public health and policy responses were primarily focused on secondary prevention and treatment of NCDs in adults.^23^ However, with the increasing realization that influences in early foetal life play such a defining role in NCDs, there has been increased attention placed on the health of both the mother and child, including the establishment of a World Health Organisation commission looking specifically at ending childhood obesity.^24^ With this change of emphasis in mind, the 2014 Gravida Summit was convened in Auckland, New Zealand to examine how early-life research programs can most strategically investigate the mechanisms, risk factors and interventions necessary for reducing the risk of childhood obesity and related diseases within New Zealand and elsewhere. New Zealand exemplifies a priority setting in this area. The recent New Zealand Government report on improving child health outcomes, particularly those relating to nutrition, obesity and NCDs, emphasized the importance of interventions at critical phases of early life such as before conception, during pregnancy, weaning and throughout early childhood.^25^

With research initiatives across New Zealand and with strong international connections, Gravida is a Centre of Research Excellence, established with New Zealand government funding a decade ago. It fosters inter-disciplinary and inter-institutional research that focuses on early-life impacts on human growth and development. In this article, the evidence provided by leading international advisers and research experts at the 2014 Gravida Summit is outlined. During the 2014 Gravida Summit, a set of recommendations were developed to serve as the basis for an integrated approach by Gravida and the wider research sector. These recommendations will help determine which areas of research into mechanisms, biomarkers and interventions could be part of Gravida’s contribution to a comprehensive early-life research strategy to fight childhood obesity and related NCDs not only within New Zealand, but also internationally.

## Strategic event

Over 140 experts, researchers and clinicians from New Zealand and other countries attended the 2-day Gravida Summit held at the University of Auckland, New Zealand on June 9–10, 2014. The speakers at the Gravida Summit are listed at the end of the document. In addition to investigators from New Zealand, speakers were from Europe (United Kingdom and Ireland), North America (Canada and United States of America), Asia (Singapore and China) and Australia. The international experts, all of whom are members of Gravida’s International College of advisors, along with New Zealand researchers, presented updates on recent basic and clinical research and best clinical practices in a series of seminars. The seminars were followed by workshops that debated and discussed ideas leading to the formation of recommendations for the future direction of the Gravida research program within three broad areas that reflect the main research themes within Gravida: mechanisms, biomarkers and opportunities for intervention. Gravida brings together biomedical, clinical and animal scientists from across New Zealand and around the world. It is important to note that a biomedical bias among the membership and international advisors is inevitably reflected in the Gravida Summit discussions and recommendations.

This article is a synthesis of a series of presented papers and moderated discussions. The framework of the article is designed to provide a coherent summary of the parallel workshops during the Gravida Summit. The recommendations presented in Table 1 reflect the need not only within the Gravida community, but also the wider *Developmental Origins of Health and Disease* (DOHaD) community. The recommendations in this article include an increase in the robustness of research into the biological processes at play, the creation of an irrefutable chain of evidence, a focus on prevention strategies (in particular the promising area of biomarkers for non-invasive risk screening) and an investment in more rigorous, system-wide evaluations of interventions that focus on both the health of mothers and children.

**Table 1:** Recommendations from the 2014 Gravida Summit

Mechanisms
1. Models systems need to be simple, tractable, reproducible and robust so that they can answer questions appropriately. Suitable models may be in cells, organisms or computational
2. Research on mechanisms must recognize the complexity of the situation and attempt to integrate the multiple levels involved. Theoretical modeling must be closely linked to the empirical research and vice versa
3. When investigating a mechanism, the ‘chain of evidence’ needs to be understood in terms of the fundamental process and biological plausibility
4. Research on mechanisms needs to be outcome focused and have measures of success
Biomarkers
5. Facilitating access to robust, high-quality biobank samples that are well phenotyped and carefully curated. Current cohorts should be harmonized, and international collaborations exploited
6. Biomarkers must be considered in the correct clinical and biological context
7. Pregnancy is a stress test, and pregnancy outcome is itself a biomarker of long-term risk of obesity and metabolic disease – research strategies and interventions need to capitalize on this realization
8. Biomarkers must be validated appropriately, including different populations and through making comparisons against all subjects without disease rather than ‘wholly uncomplicated healthy’ cases
Interventions
9. Undertaking and reporting animal intervention studies in accordance with modified ARRIVE guidelines is essential to increase reliability, reproducibility, translation and to facilitate meta-analyses
10. A continued focus on reverse translation is essential – mechanistic studies must inform intervention studies, but in turn results from intervention studies must continue to inform further mechanistic studies
11. Intervention studies must consider the short- and long-term health of the mother and offspring, as well as the inter-birth interval, the mother’s potential future pregnancies and trans-generational effects in offspring
12. Better communication of intervention study results to the public and to policy makers is imperative
13. To achieve a shift from theory to practice requires a systems implementation approach, involving stakeholders/caretakers/experts in local ‘in-need’ populations from the early stages of intervention trials through to implementation

## Areas of focus

### Mechanisms

Insults during foetal life can influence adult health through various mechanisms, including developmental plasticity, foetal programming and epigenetics. For future understanding of NCDs, it is vital that research efforts are directed at understanding and clarifying these complex mechanisms that underlie early-life exposures and later-life consequences. Although numerous animal^26^ ^–^ ^31^ and human^11^ ^,^ ^32^ ^,^ ^33^ studies have demonstrated that influences in early life have consequences for the rest of an individual’s life and into subsequent generations, the precise mechanisms mediating these effects are largely unresolved.^8^ In most cases, there is little knowledge as to how the environmental signal results in a molecular change in a particular organism and then whether this molecular change has any consequence on later-life phenotype.^8^ The absence of mechanisms underlying phenotypic plasticity is a barrier to policy and clinical uptake of DOHaD ideas.

The selection and manipulation of model systems (cells, organisms, populations or computational models) that are appropriate for a particular research question are fundamental to any research into the mechanisms underlying the plastic response observed during early life (Recommendation 1, Table 1). Models overcome many of the inherent problems associated with studying programming in humans by allowing some control of complex systems; yet in the absence of independent replication, there are still large gaps in our knowledge. It is essential that the field move towards standardized model systems wherever possible. These models systems should be tractable and reproducible in different settings, with accurate measurement of exposure(s), molecular and other hypothesized mediating biomarkers and phenotypic outcomes (R. Saffery, personal communication, 9 June 2014). Examples presented at the 2014 Gravida Summit illustrated how an early-life influence may not necessarily lead to a disease phenotype in later life, but rather may lead to an increased susceptibility to disease in response to a second insult or trigger (S. Davidge, personal communication, 9 June 2014). For example, rats exposed to hypoxia prenatally and subsequently fed on a high-fat diet had increased susceptibility to myocardial ischaemia, whereas those fed on a normal diet did not.^34^ This study reflects the complex and interactive nature of plasticity and indicates that later-life consequences (e.g. myocardial infarction) may not always be measurable without a second insult (e.g. an obesogenic nutritional environment).

Researchers must recognize that there are unavoidable complexities surrounding the mechanisms of plasticity and that these complexities need to be considered in context and with a broad approach that is integrated at multiple levels. For example, empirical research into mechanisms should be supported by collaboration with modelling, and *vice versa* (Recommendation 2, Table 1). At the 2014 Gravida Summit, examples of mathematical modelling of phenotypic plasticity were provided to illustrate their potential to provide direction for empirical research (H. Spencer, personal communication, 10 June 2014). Conversely, empirical research data should be provided to modellers in order to further understand biological output (P. Dearden, personal communication, 10 June 2014). Such collaborations and multiple-disciplinary initiatives will allow for the production of the essential ‘chain of evidence’ that links early-life influences and later-life consequences.

Establishing a ‘chain of evidence’ is critical for understanding how an early-life environmental insult may cause a stable molecular change (e.g. epigenetic) to be established and maintained in the genome that subsequently leads to a phenotypic consequence (Recommendation 3, Table 1). In humans, this requires the establishment of cohort studies with extensive environmental exposure information and longitudinally sampled biospecimens. The Peri/postnatal Epigenetic Twins Study (PETS)^35^ was presented at the 2014 Gravida Summit as an example of such a study. PETS is an Australian longitudinal study that has followed human monozygotic and dizygotic twins from prenatal stages of life to 18 months of age.^35^ Gene expression and DNA methylation data from samples taken throughout this time period have shown that twins exhibit a wide range of epigenetic discordance at birth arising from both genetic and environmental influences *in utero*.^36^ In addition, during the early-life period, one-third of the epigenome was significantly remodelled. Within-pair birth weight discordance at birth correlated with epigenetic discordance in genes associated with lipid metabolism. Such evidence supports an epigenetic mechanism for DOHaD and obesity, though replication is needed. This study also provided supporting evidence linking a specific maternal exposure in pregnancy (smoking) to stable postnatal changes in the *AHRR* gene methylation profile in children.^37^ Exposure to smoking *in utero* now represents the most replicated example of environmentally mediated epigenetic change in children. By following PETS children and similar birth cohorts over coming years, the evidence linking specific epigenetic changes in early life to later phenotypic consequences is likely to become more compelling.

In addition to measuring epigenetic changes that accompany early-life influences, researchers must also provide evidence that the correlation between phenotype and epigenetic change has a biologically functional role rather than being purely associative.^38^ To sort causation from correlation, testable systems and new technologies must be available that allow for the specific manipulation of epigenetic mechanisms. By manipulating epigenetic mechanisms at specific genomic sites, it will be possible to determine whether epigenetic changes are causative rather than simply correlated with the phenotype. By removing these gaps in the ‘chain of evidence’ and providing a more complete conceptual understanding of the mechanisms underlying plasticity, the research scientist and clinician will be able to substantiate health claims. This substantiation of health claims will be a key step towards the acceptance of DOHaD into public health and clinical practice.^39^

Importantly, research on mechanisms needs to be outcome focused (Recommendation 4, Table 1). Understanding the mechanisms involved in the ‘chain of evidence’ is the best way of knowing when and how interventions in clinics will be effective.^10^ Such knowledge will inform the development of potential drug or lifestyle interventions such as dietary supplements (including polyunsaturated fatty acids, anti-inflammatories, antioxidants, polyphenols and methyl donors) that can be used clinically.^28^ ^,^ ^40^ ^–^ ^42^ Knowledge of mechanisms ultimately provides a source of biomarkers for prognosis, diagnosis and monitoring of interventions.

### Biomarkers

Biomarkers can be defined as actively measurable, objective indicators of a particular biological or medical state.^43^ It is possible to identify biomarkers of early-life programming that predict the risk of developing a particular medical state, including obesity and related NCDs. Robust biomarkers are important in DOHaD research as they could identify individuals who are at risk of later disease due to early-life effects. The use of biomarkers for risk prediction could lead to stratification of patients, and to the targeting of interventions and potential treatments to those at highest risk.

In the field of pregnancy research, biomarker discovery efforts have yet to demonstrate significant value.^44^ ^–^ ^46^ This paucity of useful biomarkers for pregnancy and prediction of early-life programming is due to three main factors. First, the use of the candidate approach for biomarker discovery has been largely unsuccessful.^47^ Second, efforts have focused on discovering single biomarkers, when detection rates are greatly increased by combining multiple markers,^48^ particularly for complex syndromes. Third, there has been a lack of validation in distinct populations, a critical component of biomarker development.^49^ ^,^ ^50^

The first and seemingly most pressing issue for successful biomarker discovery is access to robust, high-quality biobanked samples (Recommendation 5, Table 1). Well-phenotyped biobanks are extremely important for extensive longitudinal studies. Large prospective studies, although expensive, are required to create quality biobanks suitable for biomarker discovery and for validating the clinical utility of predictive tests (L. Kenny, personal communication, 10 June 2014).^51^A common approach is needed for biobanking, which should include a set of minimum requirements for collection and phenotyping, as well as the establishment of guidelines for the economic collection of biological materials. Such requirements will facilitate comparison, comprehension and the overall usefulness of biobanks. Cohorts should adhere to relevant guidelines, such as the widely used European, Middle Eastern and African Society for Biopreservation and Biobanking Biobank guidelines^52^ or the guidelines from the Organization for Economic Co-operation and Development.^53^ Current cohorts should be harmonized, and follow the Global Pregnancy CoLaboratory model of minimum data sets.^54^ Funders should also promote cohorts in low- and middle-income countries. This approach is exemplified by the Gates Foundation^55^ and the Global Alliance to Prevent Prematurity and Stillbirth,^56^ whose promotion of cohorts in low- and middle-income countries was highly commended at the 2014 Gravida Summit. The SCOPE (Screening for Pregnancy Endpoints) study was also highlighted as an exemplar of a high-calibre, well-phenotyped pregnancy biobank.^57^

Further, the value of a biomarker increases when it is placed in the correct clinical or biological context (Recommendation 6, Table 1). Whether a biomarker is used for the prediction of risk, or for the assessment of an intervention, is key to its interpretation.^58^ Although biomarkers that accurately predict risk of disease do not necessarily need a functional story (I. Morison, personal communication, 10 June 2014), it was agreed that biomarkers were stronger if they could be related to a particular biological pathway or could be linked to a specific therapy. Although individual prediction for common human diseases is not practical at this stage, it is possible to identify groups who are at greater risk of disease.^59^

In addition to using the correct context for biomarkers, it is also critical to take a ‘whole biology’ approach to biomarker discovery rather than looking at biomarkers in isolation. With the rapidly advancing platforms for genome-wide analysis and the increasing number of ‘omics’ fields, it is essential to integrate these platforms and fields in order to build up sufficient context for biomarkers (P. Baker, personal communication, 9 June 2014). Although genome-wide association studies have successfully identified loci associated with increased risk of NCDs, including obesity,^60^ the overall effect remains small and the predictive power obtained from genetic data is poor. Epigenome-wide association studies may offer more promise,^61^ since epigenetic biomarkers offer advantages over traditional genetic variants. For example, epigenetic modifications are potentially reversible, can be modulated by relatively straightforward interventions and reflect plasticity in developmental processes.^62^ ^–^ ^64^ However, it is clear that synergy between these ‘omic’ strategies needs to be achieved in order to optimize information and efficacy of approaches to biomarker discovery.

Importantly, the best pregnancy biomarker to predict long-term risk of obesity and other NCDs is the pregnancy outcome itself (Recommendation 7, Table 1). Pregnancy is a stress test; it exposes pre-pregnancy clinical complications. Although this predictor has been known for years,^65^ it has not been acted upon. Research efforts need to capitalize on the potential of pregnancy outcome to be a biomarker for the long-term health outcomes of both mother and baby. A more considered approach to longer-term follow-up is required. For example, when a woman delivers a preterm baby, both she and her child are at higher risk of developing life-long health complications such as obesity and heart disease.^65^ Investigators and practitioners need to translate the opportunity to use pregnancy outcome as a marker of long-term risk of obesity and metabolic disease into research strategies and clinical care.

Lastly, validation is key to successful biomarker discovery (Recommendation 8, Table 1). The lack of success of many biomarker strategies is attributable to a lack of robust validation methods.^50^ In validation experiments, comparisons need to be made against all subjects without disease rather than against ‘pure uncomplicated healthy’ cases. For example, women who develop pre-eclampsia need to be compared with all women who do not have pre-eclampsia, rather than to women whose pregnancies are wholly uncomplicated. For disease risk, the value of biomarkers needs to be confirmed in different populations (A. Sheppard, personal communication, 9 June 2014). For example, poor nutrition *in utero* is associated with increased fat mass in human offspring and hyper-methylation at a specific CpG site in the promoter of the *RXRA* gene.^62^ Interestingly, the *RXRA* gene is important in determining the ‘overweight’ phenotype. This finding was validated in three distinct populations, and thus in humans, poor nutritional status *in utero* is associated with increased fat mass and the epigenetic status of a phenotypically relevant gene in the offspring.^62^ Validation was a critical step that led to this biomarker discovery success.

### Interventions

As the obesity and NCD epidemic has progressed, the need for timely and effective interventions to reduce risk and incidence has concurrently increased. It is now clear that lifestyle interventions in adulthood are largely ineffective, and the best chance to intervene effectively is early in life, specifically from the pre-pregnancy period through childhood.^5^ It is important to ascertain that early life interventions are effective, and to ensure that they do not cause harm. Throughout the workshop, the term ‘intervention’ was used broadly to encompass any strategy (pharmacological, nutritional, behavioural, etc.) to decrease obesity and NCD risk and/or incidence in individuals and populations.

Although a number of experimental paradigms in animals have proven reversibility of programmed obesity and related disorders, a meta-analysis-type approach is nearly impossible. The range of models and species used, different windows of exposure to either the initial environmental insult or the treatment/mode of intervention, and incomplete reporting of animal numbers and outcomes make assessment of bias very difficult.^66^ Although it is well established that a wide range of early-life insults can lead to increased risk of later obesity and NCD phenotypes, closer alignment of animal models where possible would make pathways to mechanistic insights and efficacy of treatment modalities more effective. Further, adherence to the ARRIVE guidelines aimed at improving the quality of reporting of animal experiments, similar to the CONSORT guidelines for human intervention trials, should increase quality and translatability between animal studies and facilitate meta-analyses similar to those performed on human data (Recommendation 9, Table 1).^67^

There are significant challenges associated with translating interventions in animal experiments to human trials. Even when an intervention approach does seem to evoke uniform responses, such as the alteration in the regulation of leptin in the early life period that results in metabolic compromise in offspring, which has been shown by a number of groups across a range of species, it is unclear how the response translates to the human.^28^ Moreover, it is important to determine whether particular challenges/interventions in animal models mirror the human situation. For example, during pregnancy in rodents, a 50% global reduction in maternal nutrition leads to offspring with increased body fat, hypertension, endothelial dysfunction and an increased inflammatory state.^68^ ^–^ ^70^ These effects are ameliorated by growth hormone treatment in the pre-weaning period, but the relevance of these outcomes to humans is unproven.^68^ ^–^ ^70^ A further issue facing investigators is that the phenotypic effects of a maternal insult on the offspring may not become evident until the offspring itself is challenged or there is a ‘second hit’ including a post-weaning obesogenic diet (M. Wlodek, personal communication, 10 June 2014).^71^ ^–^ ^73^ A ‘one size fits all’ interventional approach is likely to be unrealistic because interventions can have detrimental effects in cases where the intervention is not needed or warranted, thus reinforcing the need for effective biomarkers. Leptin and taurine treatments that are beneficial to animals whose mothers were challenged during pregnancy have adverse effects in unchallenged animals.^28^ ^,^ ^74^ Finally, in programming and intervention studies, a clear pattern of sexually dimorphic effects has emerged, but why male and female offspring respond differently to maternal challenges is not completely understood.^68^ ^–^ ^71^ In summary, although reversal of programming has been shown in a number of experimental animal models, translation to the clinic of interventions involving hormones or other compounds that may be harmful to individuals not at risk may be difficult or impossible. However, the information from animal intervention studies, perhaps in combination with biomarker discovery, will inform as to the mechanisms involved and may lead to interventions that are more uniformly applicable, such as nutritional interventions, or to a personalized approach. A continued focus on reverse translation is essential; mechanistic studies must inform intervention studies, but in turn results from intervention studies must continue to inform further mechanistic studies (Recommendation 10, Table 1).

Translation between different human studies also presents potential difficulties as the effectiveness of an intervention may depend on the population and context. For example, an intervention that is effective in a Chinese population living in Singapore may not be effective in a New Zealand Māori population.^75^ Further, within a given country, different groups of the population may need to be considered independently. A pre-pregnancy intervention that is effective in New Zealanders of European decent may not be feasible and/or effective in New Zealand Māori due to cultural differences, lifestyle, and access to resources and healthcare, etc. The dynamic and complex aetiology of obesity, which includes multiple determinants that interact with each other (including the developmental environment), requires treatment to be tailored to the in-need population, identification of the most potent early drivers of obesity and its complications, and the development and use of multi-setting, multi-component life course interventions; that is, ‘what works, for whom, and under what circumstances’ (M. Gillman, personal communication, 9 June 2014).

Given the impact that pre-pregnancy obesity, obesity during pregnancy, excess gestational weight gain and gestational diabetes has on maternal and offspring short- and long-term health, it is of interest to intervene as early as possible. However, the optimal intervention window is unclear.^20^ ^,^ ^76^ Pre-pregnancy intervention might result in the greatest effect, but may have significant cost implications and will be problematic in populations where high percentages of pregnancies are unplanned. Pregnancy is considered a pragmatic intervention time as mothers may be more willing to change for their unborn child than for themselves; however, the degree to which women actually change pre-pregnancy and during pregnancy is not as great as may be expected.^20^ ^,^ ^76^ An important consideration is that an intervention during pregnancy will affect not only the mother and baby, but also the mother’s future pregnancies and offspring. Intervention studies must consider the short- and long-term health of the mother and baby, as well as the inter-birth interval and the mother’s potential future pregnancies (Recommendation 11, Table 1). After pregnancy many women do not return to their pre-pregnancy habitus, which may predispose them to increased risk of obesity and diabetes later, and that may affect subsequent pregnancies. In addition, the best window for intervention may not be the same for all pregnancies, and will likely vary between countries, between ethnicities within countries, and between cultures.

Moving forward, it remains to be determined how to define what makes an intervention successful, and how successful interventions can best be implemented. For example, are interventions enforced with policy more effective than those reliant on individual compliance? Better communication of intervention study results to the public and to policy makers is imperative if these questions are to be addressed in future intervention strategies (Recommendation 12, Table 1). Governments want utilitarian value in research, with visible outcomes, interventions that target locally important conditions, easy implementation, easy enforcement through guidelines and policy, and importantly, the intervention must do no harm (Y.S. Chong, personal communication, 10 June 2014). To achieve a shift from theory to practice requires interdisciplinary, collaborative group work and systems approaches, and it is important to involve stakeholders/caretakers/experts in the local ‘in-need’ populations from the early stages of intervention trials through implementations (Recommendation 13, Table 1). Studying whether an intervention is effective is very different from successfully implementing it publicly, and the implementation feasibility and strategy must be considered early in the intervention trial. Although complicated and challenging, intervention studies must be informed by mechanistic research, and must focus on what can be changed in the obesity/NCD battle (the developmental environment), not what cannot be changed (adult lifestyle), and how to most effectively and successfully intervene in affected populations (P. Gluckman, personal communication, 9 June 2014).

## Conclusions

It is undisputed that obesity and related NCDs place a large burden on the public health system, economy and society in New Zealand.^77^ Mounting evidence indicates that obesity is a preventable and modifiable risk factor for many of the NCDs.^78^ Although it has become clear that there is no single solution to obesity and a multifaceted, whole-of-society approach is needed,^79^ Gravida’s early-life research program is ideally placed to be involved as part of this collective, systems approach to solving issues relating to obesity and NCDs.

The 2014 Gravida Summit was an opportunity for national and international experts, researchers and clinicians to discuss the mechanisms, biomarkers and interventions necessary for reducing the risk of obesity and NCDs within New Zealand. Stimulating discussion and debate focused on narrowing and defining specific areas of research that would best contribute to investigating the early-life events that lead to obesity. There are many serious questions that remain in this area of DOHaD research, which provide opportunities for scientists to translate knowledge into solutions.

Summit attendees established that combining research and clinical approaches to identify mechanisms, biomarkers and intervention strategies will help direct focus on maternal nutrition, obesity and associated adverse outcomes in offspring. Although previous research, including that from Gravida, has shown that early-life exposures influence the risk of obesity and NCDs later in life,^5^ ^,^ ^6^ the key conceptual component is that the early-life experience does not directly lead to obesity, but instead it alters the sensitivity of the offspring to develop obesity in an obesogenic environment.^80^

Increased knowledge about the early-life mechanisms and biomarkers of obesity and related NCDs will enable targeted interventions to reverse the rising incidence of these diseases. Robust cohorts will enable optimal use of biomarkers to directly predict long-term human health.^62^ Interventions need to occur at critical phases such as before conception, during pregnancy, weaning and through early childhood. The translation of knowledge into prevention and intervention strategies is also critical. Targeting healthy development before, and during, pregnancy and childhood will translate into advancements in public policy and clinical practices, and will lead to strong partnerships with public health services, the nutrition industry and academic medicine. A key goal is to develop interventions that advance our human capital and ensure a healthier and productive New Zealand, which can ultimately translate across the globe.

The thought-provoking discussion and debate at the 2014 Gravida Summit highlighted the unique energy and ability of Gravida investigators and their multi-disciplinary ventures. Such discussion would be further enhanced by the inclusion of additional investigators with expertise in disciplines such as research ethics and health economics; this will be addressed in future Gravida summits. Nevertheless, the series of recommendations that were formulated highlight many of the important issues that need to be addressed to tackle New Zealand’s obesity epidemic. These recommendations created a clear direction for aligning human and animal research with clinical approaches in order to combat obesity and its related diseases.
